# Past medical history of tumors other than meningioma is a negative prognostic factor for tumor recurrence in meningiomas WHO grade I

**DOI:** 10.1007/s00701-021-04780-9

**Published:** 2021-03-05

**Authors:** Annamaria Biczok, Philipp Karschnia, Raffaela Vitalini, Markus Lenski, Tobias Greve, Jun Thorsteinsdottir, Rupert Egensperger, Franziska Dorn, Jörg-Christian Tonn, Christian Schichor

**Affiliations:** 1 Department of Neurosurgery, Ludwig-Maximilians-University Munich, Marchioninistr. 15, 81377 Munich, Germany; 2 Center for Neuropathology and Prion Research, Ludwig-Maximilians-University Munich, Munich, Germany; 3 Department of Neuroradiology, Ludwig-Maximilians-University Munich, Munich, Germany

**Keywords:** Meningioma, WHO grade I, Prognostic factor, TERT promoter status, Unrelated tumor disease

## Abstract

**Background:**

Prognostic markers for meningioma recurrence are needed to guide patient management. Apart from rare hereditary syndromes, the impact of a previous unrelated tumor disease on meningioma recurrence has not been described before.

**Methods:**

We retrospectively searched our database for patients with meningioma WHO grade I and complete resection provided between 2002 and 2016. Demographical, clinical, pathological, and outcome data were recorded. The following covariates were included in the statistical model: age, sex, clinical history of unrelated tumor disease, and localization (skull base vs. convexity). Particular interest was paid to the patients’ past medical history. The study endpoint was date of tumor recurrence on imaging. Prognostic factors were obtained from multivariate proportional hazards models.

**Results:**

Out of 976 meningioma patients diagnosed with a meningioma WHO grade I, 416 patients fulfilled our inclusion criteria. We encountered 305 women and 111 men with a median age of 57 years (range: 21–89 years). Forty-six patients suffered from a tumor other than meningioma, and no TERT mutation was detected in these patients. There were no differences between patients with and without a positive oncological history in terms of age, tumor localization, or mitotic cell count. Clinical history of prior tumors other than meningioma showed the strongest association with meningioma recurrence (*p* = 0.004, HR = 3.113, CI = 1.431–6.771) both on uni- and multivariate analysis.

**Conclusion:**

Past medical history of tumors other than meningioma might be associated with an increased risk of meningioma recurrence. A detailed pre-surgical history might help to identify patients at risk for early recurrence.

## Introduction

Meningiomas are the most common primary intracranial tumors in adults. Annual incidence rate is estimated at 7.9 cases per 100,000 persons, and prevalence appears to rise with age [17]. Meningiomas are classified histologically according to the WHO into grade I–III. The most important factors associated with tumor recurrence and survival are WHO grade but also extent of surgical resection [4, 9, 11, 15, 16]. However, 20% of WHO grade I meningiomas recur after complete resection, illustrating an urgent need for further risk stratification models [9]. More recent studies have also shown an association of molecular profiles with meningioma recurrence and progression, e.g., TERT (telomerase reverse transcriptase) promoter mutation, DNA methylation profile, or loss of histone H3K27me3 [3, 20, 21]. For patients with meningioma WHO grade I and complete resection, markers prognostic of potential tumor recurrence are urgently needed to guide patient management during follow-up.

The overwhelming majority of meningiomas are considered to be sporadic. However, factors such as past medical history of tumors other than meningioma (including glioma, acute lymphocytic leukemia, prostate cancer, papillary carcinoma of the thyroid, uterus myomas, and endometriosis) but also cranial irradiation or hereditary cancer syndromes like neurofibromatosis types 1 and 2, Turner’s syndrome, and Werner’s syndrome may predispose for meningioma formation [2, 5, 7, 12, 13, 24, 28]. Similarly, within vestibular schwannomas, a shortened time to progression was observed within patients harboring an unrelated tumor disease [27]. Taken together, these findings already point to possible systemic genetic and molecular factors that play a putative role in the development of meningiomas but also in their recurrent growths.

In the present study, we describe a large cohort of histologically verified and molecularly well-defined meningiomas WHO grade I treated at a large academic cancer center with complete resection.

We thoroughly analyzed patient’s clinical characteristics, histopathological details, treatment strategies, and outcome with special focus on medical history positive for tumors other than meningioma.

## Methods

### Study design

The study was approved by the ethics committee of Ludwig-Maximilians University in Munich, Germany (approval number: 18-837). We retrospectively searched our institutional database for patients with histologically verified intracranial meningioma WHO grade I operated on between 2002 and 2016. Indications of surgical resection were symptomatic lesions, asymptomatic lesions with radiological documented tumor progression, or patients preferred wish to be operated upon an asymptomatic lesion without evidence of growth. Patients with a follow-up time of less than 6 months were excluded. For patients in which complete microsurgical resection as defined by Simpson grade I–III was provided, a comprehensive clinical and pathological chart review was performed. Further stratification into Simpson grades was not performed because results from our group showed poor significance of intraoperative estimation of the extent of resection. In contrast, postoperative imaging methods such as MRI or supplementary DOTATATE PET-CT scans showed a significantly higher reliability in the assessment of a tumor remnant [26]. Records were searched for demographics and clinical findings particularly including past medical history, neuropathological data, therapy, and outcome. Patients with known hereditary cancer syndromes (e.g., neurofibromatosis type 2) or multiple meningiomas were excluded from the study. We limited our analysis on a single solid indicator lesion. Date of death, date of last follow-up, and date of recurrence were obtained from charts filled during follow-up visits to our outpatient clinic. Recurrence-free survival was calculated as interval from first diagnosis of meningioma WHO grade I (set as date of surgical meningioma resection) until radiological recurrence. Radiological recurrence was defined as new contrast enhancement on postoperative MRI imaging obtained earliest 3 months after resection.

### WHO grading

Tumor samples from all patients included in this study were acquired through open microsurgical resection, and formalin-fixed paraffin-embedded (FFPE) specimens were used for analysis. The tumors were classified and staged according to the current WHO 2016 classification of tumors of the central nervous system. The neuropathologist was blinded for the clinical outcome data.

### DNA isolation

DNA isolation from surgical tumor specimens was performed as previously described [3]. In short, representative H&E-stained FFPE tumor slides were prepared, and tumor regions with at least 90% tumor cells were microscopically identified. DNA extraction was performed by microdissection of target regions on serial slides. Purification of DNA was performed using standard protocols. DNA quantity was determined using a NanoDrop system determining the 260/280 nm absorbance ratio.

### TERT promoter mutation analysis

Analysis of TERT mutation status was performed by amplifying the TERT promoter region including the two hotspot mutations C250T and C228T that are located − 146 bp and − 124 bp upstream of the TERT gene. We applied standard PCR protocols and direct capillary sequencing as previously described [3].

### Statistical analysis

Data is given as mean ± standard deviation of the mean if not indicated otherwise. A comparison of baseline variables between patient cohorts was performed using the X^2^ test for categorical variables, *t* test for parametric variables, and Mann-Whitney U-test for nonparametric variables. Survival function of time was illustrated via the Kaplan-Meier method (log-rank test). Multivariate analysis was performed using Cox proportional hazard regression model to estimate *p* value, hazard ratio, and 95% confidence interval. Statistical analysis was performed using a standard software package (SPSS Statistics version 25, Chicago, IL). Significance level was set at *p* < 0.05.

## Results

### Patients and clinical data

**Fig. 1:**
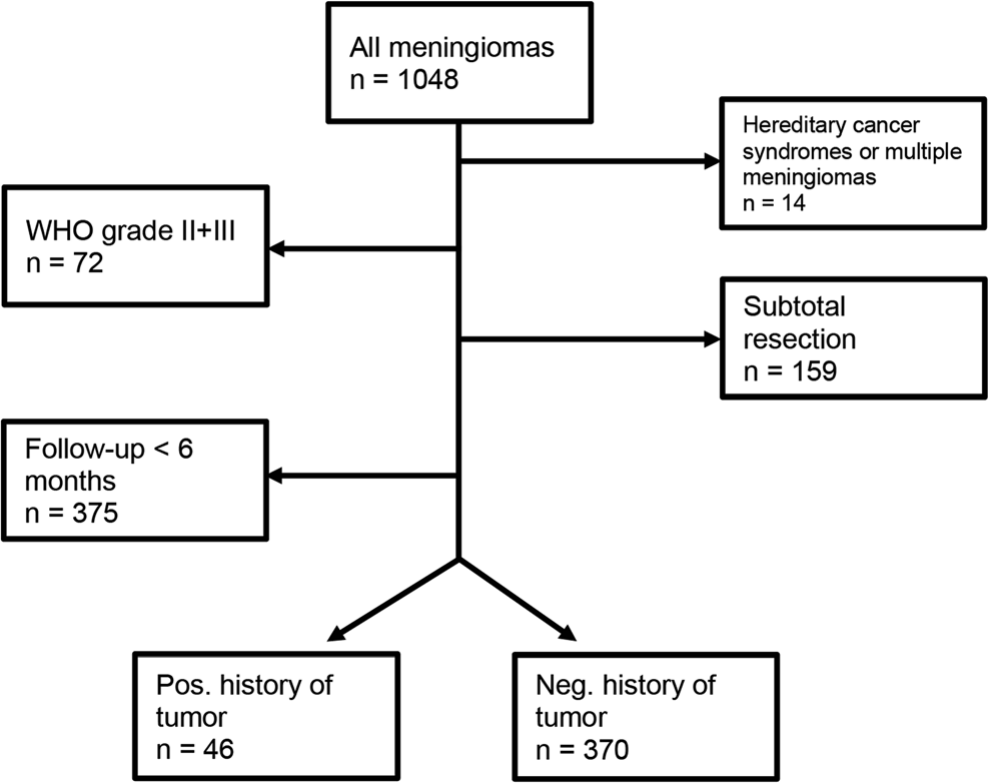
Flowchart of patient selection for inclusion in the study

Nine hundred forty-seven patients with intracranial meningioma WHO grade I treated between 2002 and 2016 were screened to meet the inclusion criteria. Three hundred seventy-five patients were excluded due to short follow-up time, and 156 patients were excluded because only subtotal tumor resection was achieved. We therefore identified 416 patients with intracranial meningioma WHO grade I who underwent complete tumor resection (Fig. 1) including 305 women and 121 men with a median age of 57 years (range: 21–89 years). Meningiomas were primarily located at the skull base (*n* = 236; 56.7%) and to less extent involved the cranial convexity (*n* = 180; 43.3%). Given that all of our highly selected patients had WHO grade I tumors and no signs of residual meningioma after resection, postoperative radiotherapy was not applied in any of our patients. The postoperative course was unremarkable in most patients.

We retrospectively assigned all 416 patients meeting our inclusion criteria into two groups based on their past oncological history (Table 1), and 46 (11.1%) patients were positive for tumors other than meningioma. We identified 19 (all females; 41.3%) out of 46 patients with breast cancer, four (8.7%) patients with ovarian cancer, three (6.5%) patients with thyroid cancer, three (6.5%) patients with uterine myoma, and 15 (32.6%) patients with other tumor entities (Table 2). TERT promoter mutation was not detected in any of the patients with a positive oncological history. Moreover, patients with and without positive medical history did not differ in terms of age, tumor location, or mitotic cell count on neuropathological meningioma analysis. Of note, predominantly female patients were positive for a positive oncological history other than meningioma (*p* = 0.005), potentially due to the high number of breast cancer patients.

**Table 1:** Patient characteristics

Clinical history of unrelated tumor disease	Yes	No	*p* value
*Total*	46 (11.1%)	370 (88.9%)	
*Median FU, months (range)*	53 (7–111)	56 (6–190)	0.910
*Median age at diagnosis, years (range)*	63 (28–83)	57 (21–89)	0.12
*Sex*			
*Female*	41 (89.1%)	264 (71.4%)	*0.005*
*Male*	5 (10.9%)	106 (28.6%)	
*Localization*			
*Skull base*	21 (45.7%)	215 (58.1%)	0.07
*Convexity*	25 (54.3%)	155 (41.9%)	
*Mitosis rate, per HPF (range)*	0 (0–3)	0 (0–1)	0.25
*Recurrence*	9 (19.6%)	34 (9.2%)	*0.034*

**Table 2:** Tumor entities

Tumor entity	*N* = 46 (%)
Breast cancer	19 (41.3)
Ovarian cancer	4 (8.7)
Leukemia/lymphoma	2 (4.3)
Renal cancer	2 (4.3)
Myoma	2 (4.3)
Lung cancer	1 (2.2)
Prostate cancer	1 (2.2)
Others	15 (32.6)

#### Outcome

**Fig. 2:**
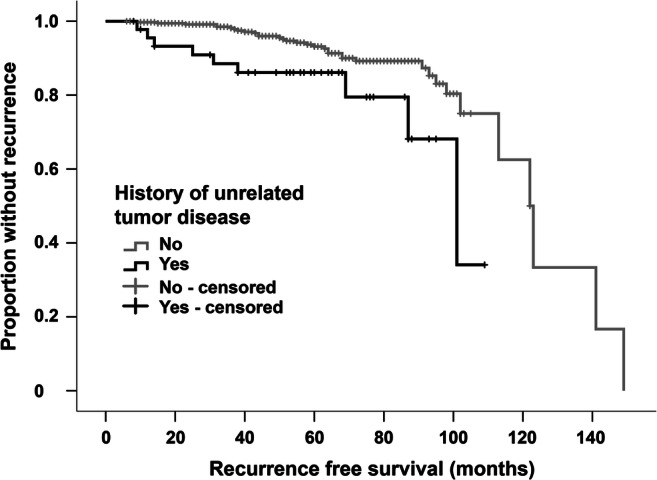
Kaplan-Meier estimates of progression-free survival (PFS) stratified according to the presence/absence of tumor disease other than meningioma

The median follow-up time after meningioma surgery was 58 months for all 416 patients (range: 6–190 months). After complete resection, the median recurrence-free survival (RFS) after complete resection of meningioma was 56 months in the entire cohort. While 9 (19.6%) out of 46 patients with a personal history of tumors other than meningioma developed tumor recurrence, only 34 (9.2%) out of 370 patients without such history did so (*p* = 0.034) (Fig. 2). Six patients suffered from a tumor progression other than meningioma, and three patients eventually died of tumor-related complications. No deaths occurred due to the progress of meningioma.

#### Multivariate analysis for prognostic factors on recurrence-free survival

Univariate and multivariate analysis was performed to identify prognostic factors of RFS for patients with WHO grade I meningiomas after complete resection (Table 3). The following covariates were included in the statistical models: age, sex, clinical history of unrelated tumor disease, localization (skull base vs. convexity), and mitosis count in meningioma samples. Interestingly, the clinical history of tumors other than meningioma was among the covariates associated with outcome (*p* = 0.004, HR = 3.1, CI = 1.4–6.8) in both uni- and multivariate analysis, with the strongest association. Male sex (*p* = 0.009, HR = 0.4, CI = 0.3–08) and tumor localization at the cranial convexity (*p* = 0.039, HR = 2.0, CI = 1.0–3.7) were also associated with shortened RFS in our cohort.

**Table 3:** Univariate and multivariate survival analysis

	Univariate analysis	Multivariate analysis		
	*p* value	*p* value	HR	95% CI for HR
*Age* (continuously)	0.725	0.907	0.999	0.974–1.024
Gender (male vs. female)	*0.009*	*0.009*	*0.405*	*0.207–0.795*
Localization (convexity vs. skull base)	*0.05*	*0.039*	*1.945*	*1.033–3.661*
History of unrelated tumor (yes vs. no)	*0.008*	*0.004*	*3.113*	*1.431–6.771*
Mitosis count (continuously)	0.984	0.602	0.879	0.542–1.427

## Discussion

Meningiomas are the most commonly encountered primary intracranial neoplasms in adults. A considerable number of meningiomas WHO grade I can be cured by surgery; however, a considerable percentage reoccurs after complete resection. Therefore, long-term tumor control remains a significant challenge in the treatment of benign meningiomas [9]. In the present study, we aimed to elucidate whether a past medical history of prior tumors other than meningioma might be an additional risk factor for recurrence after complete resection of meningioma WHO grade I [8]. To the best of our knowledge, we found for the first time that a past medical history of tumors other than meningiomas appears to be strongly correlated with the risk of meningioma WHO grade I recurrence after complete resection. Similar findings have been made among patients with radiosurgically treated vestibular schwannoma [27]. Of interest, we did not find evidence that our analysis might be confounded by other factors such as age, tumor localization (convexity vs. skull base), or mitotic cell count. Rather, the MIB index, including various cutoff values for the prediction of meningioma (WHO grade I) recurrence, is still highly controversial and therefore not included in the current WHO classification [10, 18]. In the current study, the neuropathological analysis set the emphasis on the mitotic cell index within the WHO grade I meningiomas, not considering the MIB index for proliferation. Also, other molecular and epigenetic markers in meningioma such as TERT promoter mutation, loss of histone methylation, as well as complex methylation signatures have been very recently associated with early meningioma recurrence and progression [3, 16, 20, 21]. We therefore conducted a TERT promoter status analysis in our study cohort, hypothesizing that TERT promoter mutation might link the other tumors and meningioma in our patients. However, TERT promoter mutation was absent in patients harboring both meningioma and unrelated tumor disease.

We found that most of our meningioma patients with a positive oncological history had tumors of the female reproductive tract, including breast cancer, ovarian cancer, and uterine myoma. Individuals with hereditary mutations in BRCA1 and BRCA2 are predisposed to a higher risk of breast or ovarian cancer. Lombardi et al. illustrated that the silencing of BRCA1 by hypermethylation might also play an important role in meningioma development [14]. Furthermore, a polymorphic variation within the BRIP1 is a risk factor for meningioma development and was also identified in breast cancer patients [2]. On a cautionary note, we did not find evidence of familial cancer syndromes in our patients. Tumors of the female reproductive tract as well as meningioma were shown to express high levels of estrogen and progesterone receptors on their cell membrane, which makes them both particularly susceptible to exogenous hormone levels and may explain the female predominance among meningioma patients [13]. Heightened hormonal levels have also been associated with the myoma development, and women with myomas were reported at increased risk for meningioma [28]. Genome-wide screening of potential genetic predispositions, linking the aggressive meningioma phenotype to the other cancers encountered in our cohort, was outside of our main focus. However, we believe that our study results will serve as a solid starting point for pursuing such hypothesis in future studies.

Most of our patients with a positive oncological history had malignant and rather aggressive neoplasms, which warranted prior anti-tumor therapy. Systemic chemotherapy and radiotherapy to the neuroaxis provided in survivors of childhood cancers have been shown to dramatically increase the risk of meningioma [6, 12]. In our series, two patients were receiving CNS axis radiation as a treatment for the malignancy other than meningioma; the remaining patients had no radiation of the skull and neck area. In turn, environmental and lifestyle factors such as tobacco use and excessive alcohol intake are known to be associated with a variety of cancers we have encountered, but an association with meningioma could not be established in prior studies. Although rather controversial, sex hormones, obesity, hypertension, and diabetes might be more likely to be associated with risk of meningioma development [1, 22, 23]. We did not control for such factors, and we cannot rule out the possibility that such markers might have confounded our analysis. Whether positive personal history for tumors other than meningiomas is a completely independent risk factor for meningioma recurrence remains therefore to be shown. Other limitations of our study may include its retrospective design and the rather small number of patients with a positive oncological history. Given the limited sample size, we were not able to perform an elaborated subgroup analysis for outcome among patients with a positive oncological history.

In the current study, we demonstrated that male sex might be associated with significantly shorter RFS. Sex-dependent differences in the prognosis of meningiomas have yet been reported with less favorable outcome among male patients [19]. One possibility is the progesterone-receptor independent growth of meningiomas in male patients. However, it had been shown that the progesterone receptor is only expressed on non-dividing meningioma cells. Thus, further genetic alterations that might provide an explanation have yet to be elucidated [25].

Collectively, our data show that a past medical history positive for tumors other than meningioma is associated with less favorable recurrence-free survival in meningioma patients after complete resection. These results may be useful to guide personalized patient management during the postoperative follow-up period, and a closer follow-up might be particularly recommended in patients with an oncological past medical history. Further studies may identify systemic genetic alterations or etiological risk factors underlying both meningioma development and formation of independent tumors.
